# Surface Curvature Effect on Dual-Atom Site Oxygen Electrocatalysis

**DOI:** 10.1021/acsenergylett.3c00068

**Published:** 2023-02-07

**Authors:** Ritums Cepitis, Nadezda Kongi, Jan Rossmeisl, Vladislav Ivaništšev

**Affiliations:** † Institute of Chemistry, University of Tartu, Ravila 14a, 50411 Tartu, Estonia; ‡ Department of Chemistry, Center for High Entropy Alloy Catalysis, University of Copenhagen, Universitetsparken 5, 2100 Copenhagen, Denmark

## Abstract

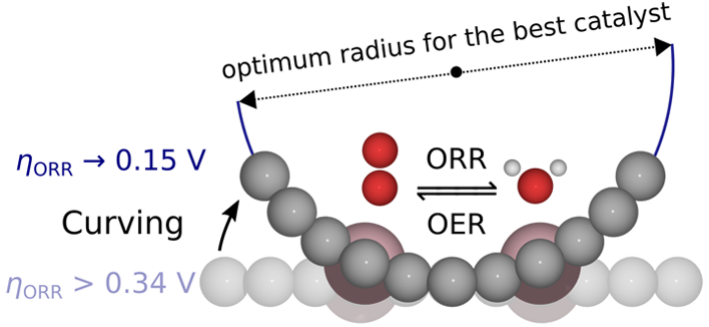

Improved oxygen electrocatalysis is crucial for the ever-growing energy demand. Metal–nitrogen-carbon (M–N–C) materials are promising candidates for catalysts. Their activity is tunable via varying electronic and geometric properties, such as porosity. Because of the difficulty in modeling porosity, M–N–Cs with variable surface curvature remained largely unexplored. In this work, we developed a realistic in-pore dual-atom site M–N–C model and applied density functional theory to investigate the surface curvature effect on oxygen reduction and evolution reactions. We show that surface curving tailors both scaling relations and energy barriers. Thus, we predict that adjusting the surface curvature can improve the catalytic activity toward mono- and bifunctional oxygen electrocatalysis.

Metal–nitrogen–carbon (M–N–C) catalysts are promising candidates to reduce the reliance on expensive platinum-group metals and achieve reliable activity for oxygen reduction and evolution reactions (ORR/OER).^1−5^ The main feature of M–N–C catalysts is their porous structure with single-atom sites, providing an adjustable site environment and efficient atom usage.^6,7^ Density functional theory (DFT) has highlighted the principal limitations in the development of new M–N–C catalysts.^8,9^ For ORR and OER, the main limitation is the scaling relation for two key intermediates, OH and OOH.^10−14^

Figure 1 illustrates the evolution of M–N–C models that address the OH–OOH scaling relation at the DFT level. The simplest model is a single-atom site catalyst composed of four nitrogen atoms surrounding a metal atom (MeN_4_) atom embedded within a graphene layer (Figure 1a). Changing the metal center in the MeN_4_ site simultaneously and proportionally varies the adsorption energies of all intermediates; thus, it is limited by the OH–OOH scaling relation.^15^

**Figure 1:**
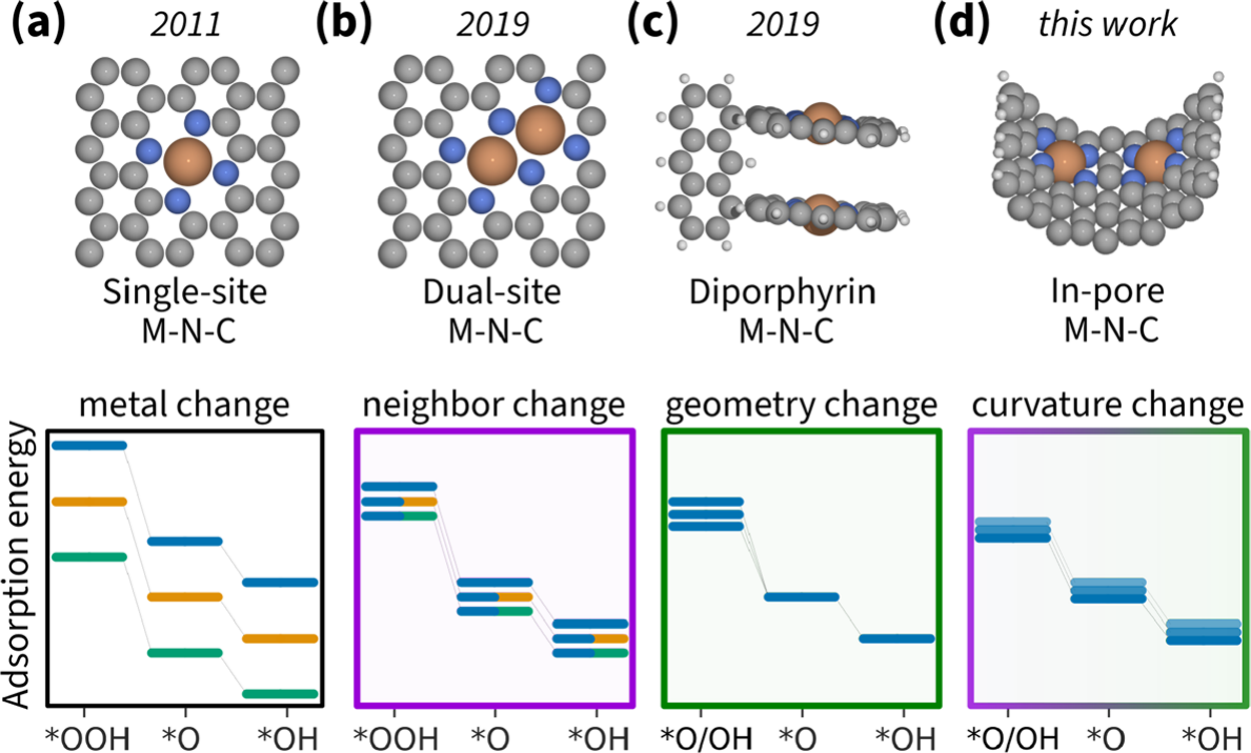
Timeline of the M–N–C model development. The adsorption energies of intermediates vary with changing (a) M in the flat single-atom site model,^15^ (b) second M in the flat dual-atom site model,^16^ (c) geometry of the diporphyrin model,^23^ and (d) surface curvature of the in-pore model. Line color indicates a different M, line transparency indicates distinct geometry.

A more recent improvement on the MeN_4_ model is the Me_2_N_6_ model (Figure 1b)—a dual-atom site catalyst formed by four nitrogen atoms surrounding two metal atoms. Combinatorial variation of these sites provides finer control over the adsorption energy.^16^ Furthermore, the second metal atom can help dissociate O_2_ and hence enable an alternative mechanism that can potentially circumvent the OH–OOH scaling relation.^17−19^ Nevertheless, Svane et al. found that, for Co_2_N_6_, the limiting step is the OH desorption and, hence, breaking the scaling relation does not improve the overpotential.^20^

An alternative to Me_2_N_6_ is a dual-atom catalyst mimicking the cytochrome c oxidase.^21^ One such model is the diporphyrin system shown in Figure 1c—a dual-atom site catalyst that enables an alternative reaction mechanism excluding OOH and switching between scaling relations.^22−24^ Regrettably, this artificial model requires impractical atomic-level control to make a real electrode.

In this Letter, we propose a curved model with two MeN_4_ sites (Figure 1d)—an in-pore dual-atom site catalyst for which we show below that it can also switch between scaling relations while providing more precise control of all adsorption energies. This model allows studying the variation of the main intrinsic features of real M–N–C materials: intersite distance and surface curvature.

The general effects of intersite distance and surface curvature on electrocatalytic activity have been discovered just recently.^25−29^ On the one hand, the previously reported surface curvature effect mimics the well-known strain effect.^30^ On the other hand, to the best of our knowledge, the surface curvature effect that enhances dual-atom site catalysis and goes beyond the scaling relations remains unknown. Porous carbon materials, such as M–N–Cs, are ideal candidates to investigate this effect because they are inherently curved.^31−34^ A significant fraction of double-atom sites among randomly distributed single-atom sites is visible in many M–N–C materials, for example, in microscopic images.^35−37^ Hypothetically, optimizing the surface curvature and intersite distance of M–N–Cs can change the reaction mechanism and increase the OER/ORR activity.

This work presents a realistic in-pore dual-atom site M–N–C model that demonstrates how surface curvature affects the activity of ORR and OER beyond the OH–OOH scaling relation limitations.

## In-Pore Dual-Atom Site Model

**Figure 2:**
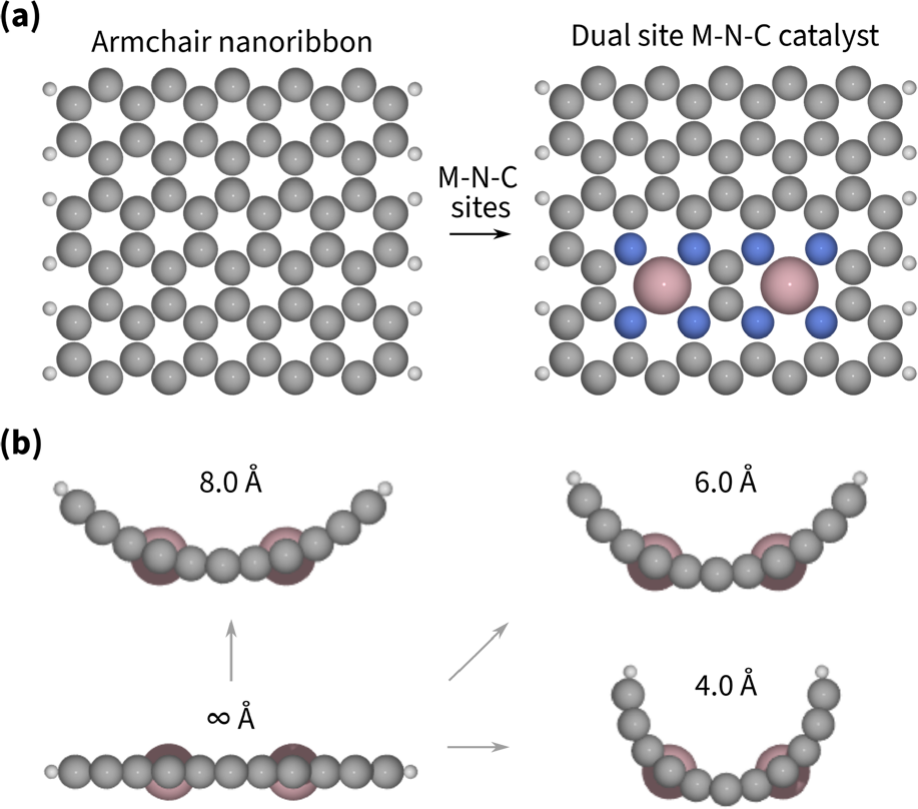
(a) Top view on constructing a dual-atom site M–N–C model from an armchair nanoribbon. (b) Side view on curving the flat dual-atom M–N–C model into in-pore models. A periodic boundary condition was applied along the pore.

The surface curvature is a parameter common for all porous carbon materials. More specifically, for nanotubes and cylindrical pores, the surface curvature is the inverse of the inner radius (*r*). Our curved models (*r* = 4, 6, 8, 8.5 Å) represent the extent of surface curvature within micropores, while the flat model (with *r* = *∞*) is a reference point for the usual adsorption behavior. To construct the model, we start with an armchair nanoribbon, introduce two MeN_4_ sites, and then curve the surface to the desired radius, as shown in Figure 2. The width of the armchair nanoribbon was chosen such that newly inserted MeN_4_ sites are away from the edges of the nanoribbon. Hence, the interactions are independent of the type of nanoribbon chosen, and the armchair nanoribbon is chosen due to convenient symmetry. We considered two models: with two CoN_4_ sites and with CoN_4_ plus a NiN_4_ site. These models are further referred to as CoCo and CoNi models, respectively. Cobalt was chosen based on existing information on optimal adsorption energies on single-site catalysts; nickel was chosen as a lesser binding site to compare the effect of the secondary site.^15^

## Surface Curvature and Adsorption Energies

**Figure 3:**
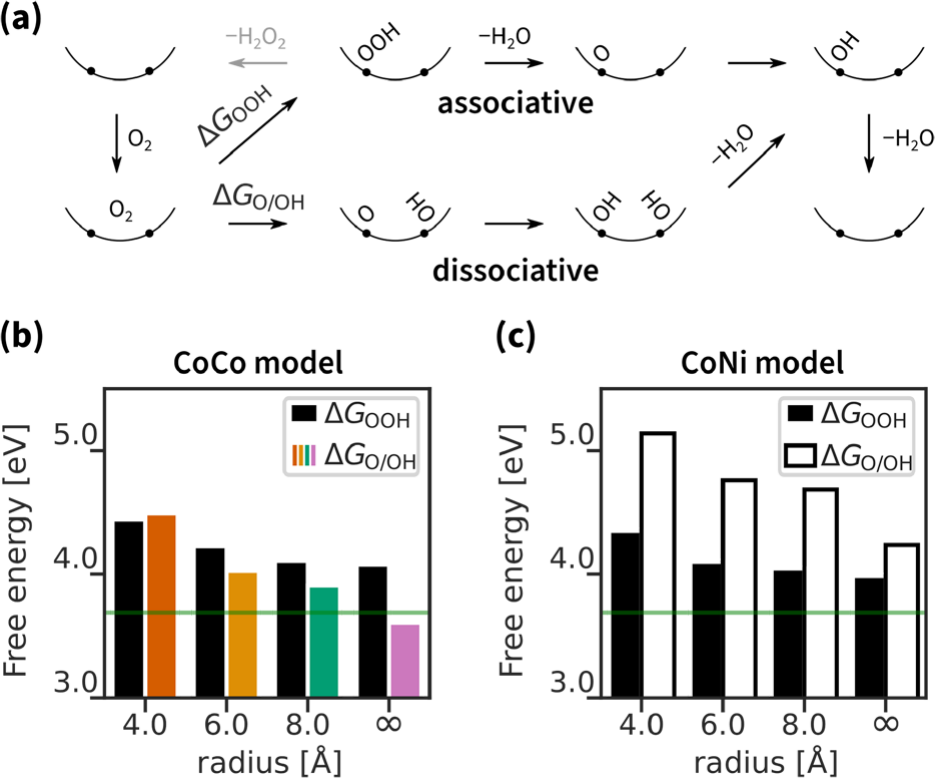
First ORR intermediate adsorption energy for associative and dissociative mechanisms: (a) schematic drawing of both mechanisms, (b) CoCo model energies, and (c) CoNi model energies. The green line indicates the ideal adsorption energy.

The surface curvature affects the adsorption energy of each ORR intermediate within both associative and dissociative mechanisms (Figure 3a, for the raw DFT energy data see Table S1). The dependence of Δ*G*_OOH_ and Δ*G*_O/OH_ on the pore radius in Figure 3b,c has two components.

First, the metal effect on adsorption energy. Black bars in Figure 3b,c indicate similar Δ*G*_OOH_ adsorption energies for CoCo and CoNi models at equal radii. That is expected for the associative mechanism due to the weak neighboring effect and the preferable strong binding of OOH to the CoN_4_ site. In contrast, the colored and white bars in Figure 3b,c indicate lower Δ*G*_O/OH_ adsorption energies for the CoCo model than for the CoNi model at equal radii. That is expected for the dissociative mechanism because of the weak binding of OH to the NiN_4_ site.

Second, the curvature effect on adsorption energy. Data in Figure 3a,b show a clear trend of decreasing adsorption energy with increasing radius. The decrease correlates with a change in electronic structure upon curving (see the density of states analysis in the Figure S3). The decrease is steeper for dissociative than for associative mechanisms because of the simultaneous adsorption of two intermediates (O and OH) rather than one (OOH). Above all, curving the CoCo model surface from flat to 8 Å adjusts the adsorption energy to the ideal value of 3.69 eV (green horizontal line). Nevertheless, in addition to reaching an ideal adsorption energy, it is more important to adjust the energy differences for efficient catalysis.

## Surface Curvature and Scaling Relations

**Figure 4:**
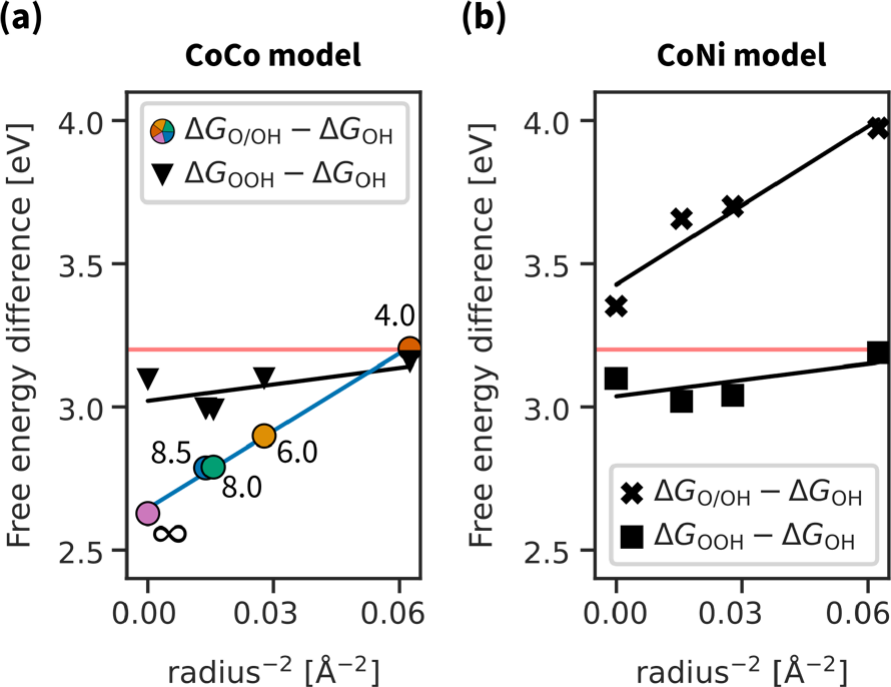
Linearized plot of free energy differences vs the intersite distance which is expressed as *r*^–2^. Using colored points as a reference, the pore radius of the CoCo model is respectively *∞*, 8.5, 8.0, 6.0, and 4.0 Å from left to right.

For the dissociative mechanism, surface curving allows adjusting the difference between the adsorption energies of two intermediates. Figure 4 shows the dependence of Δ*G*_O/OH_ – Δ*G*_OH_ and Δ*G*_OOH_ – Δ*G*_OH_ on the intersite distance, which is inversely proportional to the squared pore radius (for derivation, illustration of the quantities, and plot, see the Supporting Information, Figure S1 and Figure S2, respectively):
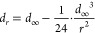
1where *d*_*r*_ is distance at radius *r* and *d*_*∞*_ is the intersite distance on the flat surface.

For the associative mechanism, the free energy difference is almost independent of the pore radius and is close to the expected OH–OOH scaling relation of 3.2 eV. On the contrary, for the dissociative mechanism, there is a clear Δ*G* ∼ *r*^–2^ linear dependence. Moreover, for the CoCo model, the switch of scaling relations allows going below 3.2 eV and approaching the ideal difference of 2.46 eV. However, that is not enough for an ideal catalyst, as the dissociative reaction overpotential also depends on O and OH/OH intermediates in addition to OH O/OH.

## Surface Curvature and Climbing of Overpotential Volcanoes

**Figure 5:**
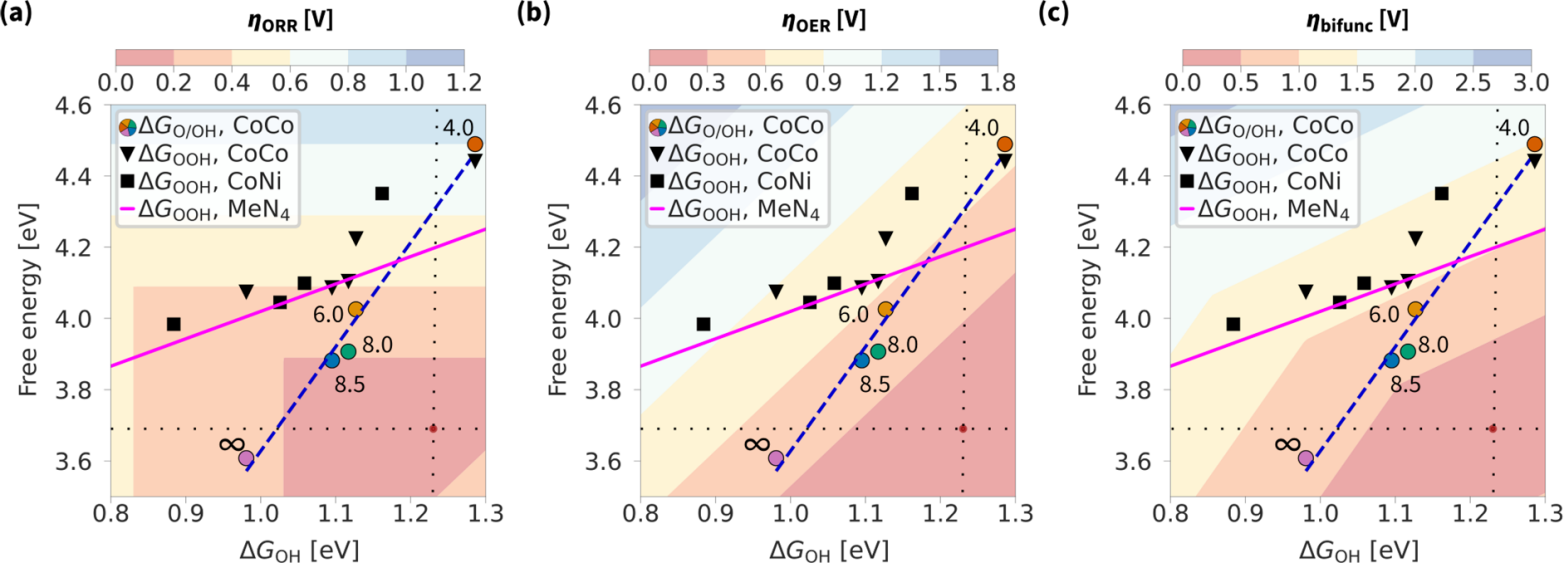
3D overpotential heatmaps (volcanoes) for (a) ORR, (b) OER, and (c) bifunctional overpotentials. Using colored points as a reference, the pore radius of the CoCo model is respectively *∞*, 8.5, 8.0, 6.0, and 4.0 Å from left to right. The volcano tops (η = 0 V) are marked with a dot at the crossing of thin dotted lines. Plot with Bayesian error estimation is shown in Figure S5.

In Figure 5, we plot the 3D ORR overpotential heatmap (volcano)^23^ and further generalize it to OER and bifunctional overpotentials. The ORR overpotential in terms of intermediate adsorption energies is defined as
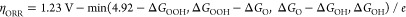
2and for OER
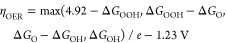
3with bifunctional overpotential η_bifunc_ = η_ORR_ + η_OER_. These expressions and the assumption that Δ*G*_OH_ ≈ 2Δ*G*_O_ define the contours in Figure 2.^22,23^

The black data points (for the associative mechanism) are situated around the comparison line, which represents MeN_4_ energies from ref (15) and obeys the OH–OOH scaling relation. The fitting of that line is shown in the Supporting Information in Figure S4.

The colored data points (for the dissociative mechanism on the CoCo model) follow a dashed line characteristic for the OH–O/OH scaling relation.^23^ With increasing pore radius, this line enters the low-overpotential region. For ORR in Figure 5a, the dashed line is only 0.15 V far from the volcano top when the pore radius is slightly above 8 Å. For OER in Figure 5b, the situation is different, as the volcano shows a steeper dropoff of overpotentials than for ORR, and the predicted radius–energy relationship does not improve the OER activity as significantly. Overall, the resulting bifunctional activity is highest, with the models having pore sizes of 8.0 and 8.5 Å.

## Surface Curvature and Overpotential of Reactions

**Figure 6:**
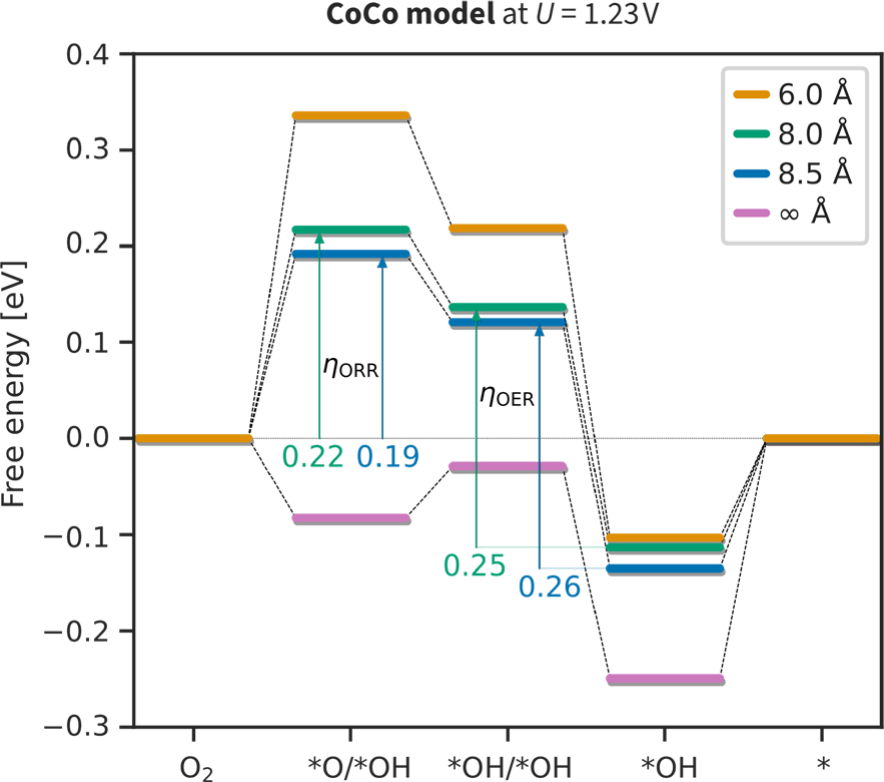
Free energy diagram for the ORR dissociative mechanism. Results were obtained at the CoCo model with variable pore radius and at an equilibrium potential of 1.23 V.

In Figure 6, we compare the overpotentials for bifunctional oxygen catalysis on a complete free energy diagram. It shows that increasing the pore radius from 8.0 to 8.5 Å decreases the η_ORR_ but increases the η_OER_. As a result, the optimal η_bifunc_ = 0.45 V is achieved around *r* = 8.5 Å.

Note that the bifunctional overpotential is significantly lower than indicated by the overpotential volcano shown in Figure 5c. More specifically, η_OER_ in Figure 5b is around 0.4 V, whether the directly calculated overpotential is 0.25 V. In general, more precise overpotential calculations (Figure 6) predict even higher activity than those given by the approximation Δ*G*_OH_ ≈ 2Δ*G*_O_ (Figure 4).

## Surface Curvature and the Dissociation Barrier

As shown above, the OOH dissociation to O/OH switches the scaling relation from OH–OOH to a more favorable OH–O/OH and theoretically increases the activity. The dissociation also excludes the 2*e*^–^ reduction to H_2_O_2_, which is known to affect the ORR selectivity for single-atom site catalysts.^38,39^ However, both activity and selectivity depend on the reaction kinetics. Data in Table 1 and Figure S6 indicate that surface curving affects a key parameter in reaction kinetics—the dissociation barrier. On a plane surface (*r* = *∞*), the dissociation is thermodynamically favorable, but a high barrier makes it kinetically impossible. On a strongly curved surface (*r* = 4.0 Å), the dissociation is thermodynamically unfavorable (due to intermediate repulsion), while the barrier is favorably low. In between, the dissociation is thermodynamically favorable, but the dissociation barrier grows with increasing pore radius as the metal distance increases. Hence, moderate curving can effectively balance kinetics (Table 1), thermodynamics (Figures 3 and 4), catalytic activity (Figures 5 and 6), and selectivity (Figure 3a).

**Table 1:** Results of Nudged Elastic Band (NEB) Calculations for the Dissociation of OOH to O/OH^a^

*r* [Å]	*G*_a_ [eV]	Δ*G* [eV]
*∞*	0.97	–0.45
8.5	0.78	–0.19
8.0	0.68	–0.18
6.0	0.51	–0.18
4.0	0.32	0.07

a Results obtained at the CoCo model with variable pore radius (*r*). *G*_a_ is the activation barrier; Δ*G* = Δ*G*_O/OH_ – Δ*G*_OOH_ is the dissociation reaction free energy.

In summary, using DFT calculations, we found that optimizing the surface curvature can reduce the ORR overpotential below 0.20 V and achieve a bifunctional overpotential as low as 0.45 V, i.e., lower than 0.74 V set by the OH–OOH scaling relation. The finding promises an advance in (bifunctional) oxygen electrocatalysis in fuel cells, electrolyzers, and air batteries.

We show that changing the curvature changes the electronic structure and intersite distance. These changes enable a dissociative mechanism and stabilize the intermediates—switching the scaling relation from OH–OOH (limiting the overpotentials to 0.37 V) to OH–O/OH (limiting the overpotentials to 0.15 V). The first way—changing the mechanism—is well-known,^23,40^ while the second one—stabilization by means of surface curving—is demonstrated for the first time.

The curvature effect remains generally unknown because curved models are rare due to their peculiar geometry and periodicity (cf. ref (41)). For example, two studies demonstrated a curvature effect for wide nanotubes, where the catalysis occurs on the outer side of the nanotube with a single-atom site.^28,42^ For such models, the change in activity was achieved by altering the electronic structure and, in the presence of single-atom sites, cannot overcome the OH–OOH scaling relation. The presented dual-atom site model has adjustable curvature while retaining periodicity along the pore (see Figure 2) and suits future studies on in-depth aspects of in-pore electrocatalysis, such as different metal centers, stabilization *via* oxophilic spectator ligands, and microkinetic modeling.^40,43^

We suggest verifying the surface curvature effect by varying the intersite distance and pore radius in M–N–C catalysts. Distribution of these two intrinsic properties of porous M–N–Cs can be regulated by the synthesis conditions. Novel M–N–C catalysts can be, in principle, made with natural curving and randomly allocated in-pore dual-atom sites, unlike nanotubes, which are synthesized with specific radii and regularly allocated out-pore single-atom sites.^44−48^ Let us note that specific care must be taken to ensure that the M–N–C catalyst is sufficiently resistant to oxidation at OER conditions.^49^ In regard to material stability, we refer readers to studies on the oxidation of carbon materials^50,51^ and carbon support for fuel cells.^52−54^

## Data Availability

Model structures, total energy values, and analysis scripts are available on the webpage https://nano.ku.dk/english/research/theoretical-electrocatalysis/katladb/surface-curvature-effect-on-oxygen-electrocatalysis/.

## Supporting Information Available

Computational details, description of the in-pore dual-atom site model (Figures S1 and S2), PDoS analysis (Figure S3), fitting of the MeN_4_ data (Figure S4), Bayesian error estimation (Figure S5), NEB analysis (Figure S6), and all binding energies used in this work (Table S1) (PDF)
