# Supplementary material for: Surface Curvature Effect on Dual-Atom Site Oxygen Electrocatalysis
Source: ACS Energy Lett. 2023 Feb 7;8(3):1330–5. doi: 10.1021/acsenergylett.3c00068 (PMC10013177; doi:10.1021/acsenergylett.3c00068)
Supplement: Supplementary file 1 — nz3c00068_si_001.pdf [file nz3c00068_si_001.pdf]

Supporting Information: Surface Curvature Effect on Dual-Atom Site Oxygen Electrocatalysis

Ritums Cepitis,† Nadežda Kongi,*,† Jan Rossmeisl,‡ and Vladislav Ivaništšev*,‡

†*Institute of Chemistry, University of Tartu, Ravila 14a, 50411 Tartu, Estonia*

‡*Department of Chemistry, Center for High Entropy Alloy Catalysis, University of Copenhagen, Universitetsparken 5, 2100 Copenhagen, Denmark*

E-mail: nadezda.kongi@ut.ee; vliv@chem.ku.dk

Computational details Spin-polarized Density Functional Theory (DFT) calculations were performed with the GPAW 22.1 and ASE 3.22.1 software in the finite-difference mode using the revised Perdew-Burke-Ernzerhof (RPBE) functional.[S1–S3] For optimization and energy calculations, 3×1×1 k-point sampling, grid spacing of 0.18 Å, at least 5 Å vacuum layer, and force minimization below 0.1 eV/Å were used. Atomic regions were treated with the PAW formalism; 9, 16, 4, 5, 6, and 1 valence electrons were included for each Co, Ni, C, N, O, and H atom, respectively, from the GPAW setups 0.9.2. A 0.3 eV correction for ∗OOH and ∗OH was applied to account for the solvation of ∗OH and ∗OOH species.[S4] Vibration and zero-point energy corrections were taken from previous studies.[S5] Dispersion correction was accounted for using the D4 method.[S6]

Model structures, total energies, and analysis scripts are available on the webpage `https://nano.ku.dk/english/research/theoretical-electrocatalysis/katladb/surface-curvature-effect-on-oxygen-electrocatalysis/`.

For the oxygen reduction reaction (ORR):

$$O_2 + 4\,(H^+ + e^-) \longrightarrow 2\,H_2O \quad E^\circ = 1.23\,V \tag{1}$$

and the oxygen evolution reaction (OER) is considered as the reverse reaction. The ORR can proceed via two mechanisms – *associative* mechanism:

$$* + O_2 + (H^+ + e^-) \longrightarrow *OOH \tag{2}$$

$$*OOH + (H^+ + e^-) \longrightarrow *O + H_2O \tag{3}$$

$$*O + (H^+ + e^-) \longrightarrow *OH \tag{4}$$

$$*OH + (H^+ + e^-) \longrightarrow * + H_2O \tag{5}$$

and *dissociative* mechanism:

$$2\,* + O_2 + (H^+ + e^-) \longrightarrow *O + *OH \tag{6}$$

$$*O + *OH + (H^+ + e^-) \longrightarrow *O + * + H_2O \tag{7}$$

$$*O + * + (H^+ + e^-) \longrightarrow *OH + * \tag{8}$$

$$*OH + * + (H^+ + e^-) \longrightarrow 2\,* + H_2O \tag{9}$$

where $*$ indicates the adsorption site.

The free energy (G) of each intermediate was calculated as:

$$G = E + \mathrm{ZPE} + TS + \mathrm{SC} \tag{10}$$

where E is the DFT energy with the D4 correction, ZPE is the zero-point energy, TS is the entropic correction, SC is the solvation correction.

The adsorption free energies (ΔG) of each intermediate were obtained for *associative* mechanism as:

$$\Delta G_{\mathrm{OOH}} = G_{\mathrm{OOH}} - G_{*} + \tfrac{3}{2}G_{\mathrm{H_2}} - 2G_{\mathrm{H_2O}} - 3eU \tag{11}$$

$$\Delta G_{\mathrm{O}} = G_{\mathrm{O}} - G_{*} + G_{\mathrm{H_2}} - G_{\mathrm{H_2O}} - 2eU \tag{12}$$

$$\Delta G_{\mathrm{OH}} = G_{\mathrm{OH}} - G_{*} + \tfrac{1}{2}G_{\mathrm{H_2}} - G_{\mathrm{H_2O}} - eU \tag{13}$$

and for the *dissociative* mechanism as:

$$\Delta G_{\mathrm{O/OH}} = G_{\mathrm{O/OH}} - G_{*} + \tfrac{3}{2}G_{\mathrm{H_2}} - 2G_{\mathrm{H_2O}} - 3eU \tag{14}$$

$$\Delta G_{\mathrm{OH/OH}} = G_{\mathrm{OH/OH}} - G_{*} + G_{\mathrm{H_2}} - 2G_{\mathrm{H_2O}} - 2eU \tag{15}$$

$$\Delta G_{\mathrm{OH}} = G_{\mathrm{OH}} - G_{*} + \tfrac{1}{2}G_{\mathrm{H_2}} - G_{\mathrm{H_2O}} - eU \tag{16}$$

where the computational hydrogen electrode (CHE) was used to calculate the energy of $(\mathrm{H^+} + \mathrm{e^-})$ at potential U as $\frac{1}{2}G_{\mathrm{H_2}} - eU$ with e being the elementary charge. $*$ in energies of adsorbed species are omitted for the sake of brevity.[S7]

In-pore dual-atom site Model Figure S1 shows our curved model of a metal-nitrogen-doped carbon (M-N-C) catalyst with two metal sites. The model variable parameters include the metal centers of active sites Me^1, Me^2, and pore curvature r (thus fixing also angle θ and metal atom distance d_r).

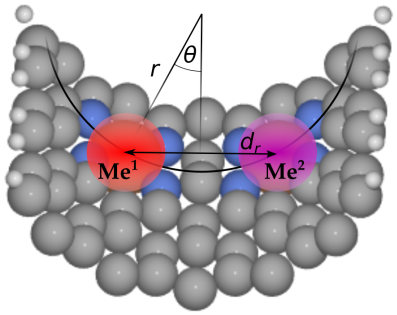

Figure S1: In-pore dual-atom site M-N-C model with two metals and pore radius (r).

Since the most crucial model descriptor is the metal atom distance, let us consider the distance between metal centers in the planar configuration as d_∞ and the metal atom distance at pore radius r as d_r. d_r can be expressed in terms of r and θ to yield

$$d_r = 2r\sin(\theta) \tag{17}$$

while the chord length equals to d_∞ and so expressing it in terms of r and θ yields

$$d_\infty = \frac{2\theta}{2\pi} \cdot 2\pi r = 2\theta r \tag{18}$$

which can be rearranged to find that $r = \dfrac{d_\infty}{2\theta}$ and so expression for d_r becomes

$$d_r = 2\frac{d_\infty}{2\theta}\sin(\theta) = d_\infty \operatorname{sinc}(\theta) = d_\infty \operatorname{sinc}\left(\frac{d_\infty}{2r}\right) \tag{19}$$

The constraint on the size of the pores (representing physical pores) imposes a condition $r > 3$ Å. The expression can be simplified further by applying Taylor expansion to give

$$d_r \approx d_\infty \left(1 - \frac{d_\infty^2}{3! \cdot (2r)^2} + \frac{d_\infty^4}{5! \cdot (2r)^4} - \cdots \right) \tag{20}$$

In the region of interest ($r > 3$ Å), only the first two terms of the expansion are necessary to approximate the function sufficiently. Hence the functional form for distance dependence on curvature can be expressed as

$$d_r = d_\infty - \frac{d_\infty^3}{24} \cdot \frac{1}{r^2} \tag{21}$$

The comparison of the analytical solution to the approximate form is shown in Figure S2.

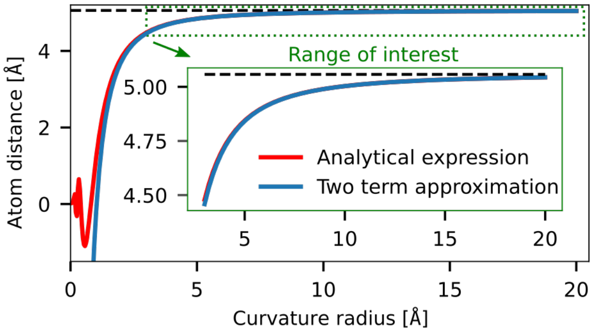

Figure S2: A plot of analytical and approximate solutions, with an inset showing the realistic region of interest.

Partial density of states of adsorbing metal

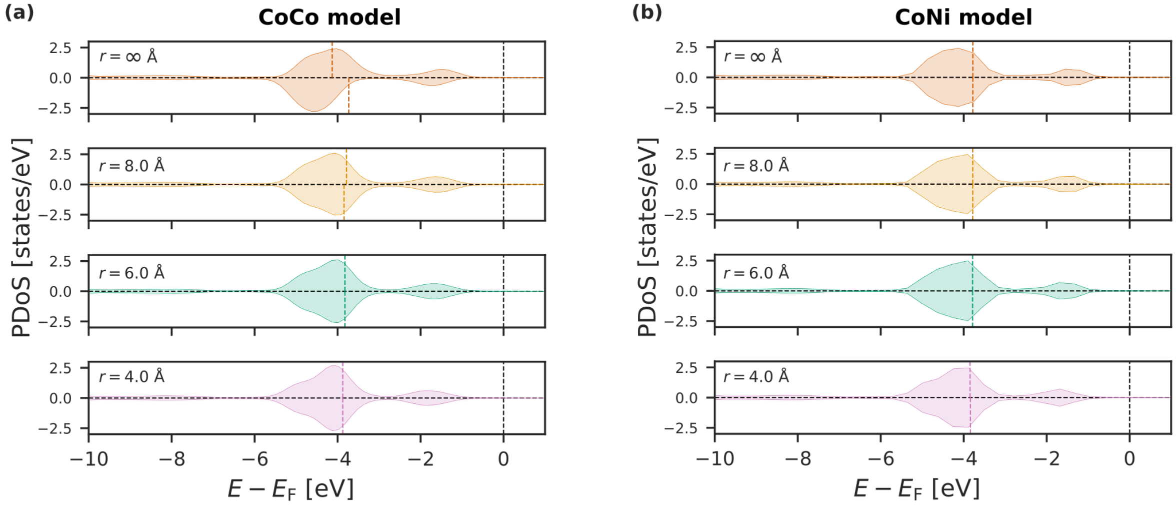

Figure S3: Partial density of states (PDoS) for the adsorbing metals in the (a) CoCo and (b) CoNi models. Colored dashed lines indicate d-band center ϵ_d. Note the general shift of ϵ_d to lower energies as r decreases, resulting in weaker adsorption of oxygenated species.

Scaling and overpotential volcano on MeN_4 analysis

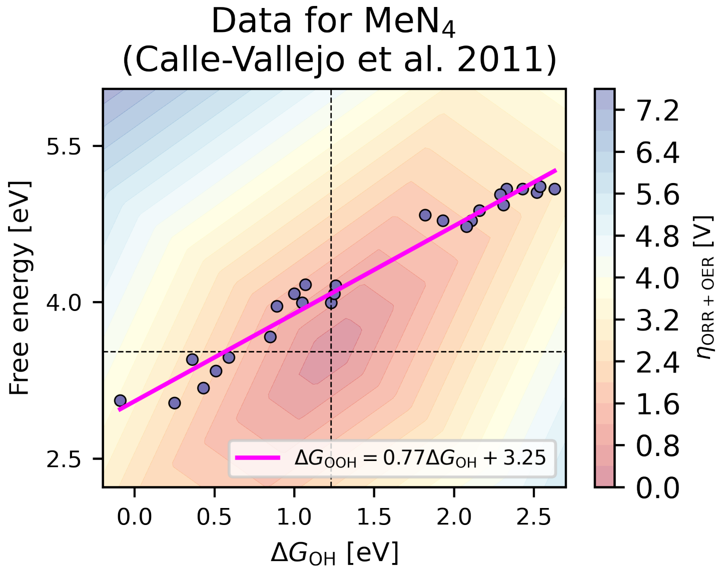

Figure S4: Construction of the magenta line shown in main article using data from reference.[S5]

Bayesian error estimation errors and overpotential volcanoes Relative adsorbate errors in terms of covariance ellipses were obtained with the Bayesian error estimation (BEE) method.[S8,S9] The BEE values were calculated for the RPBE functional with a modified GPAW code. In Figure S5, a different tilting direction of the covariance ellipses confirms the different scaling relations for the associative and dissociative mechanisms.

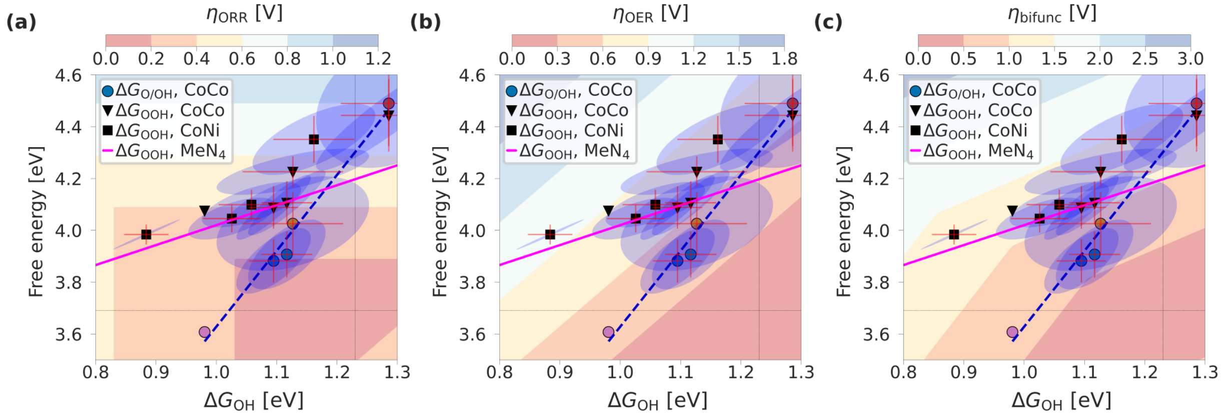

Figure S5: 3D volcanoes contour map for (a) ORR, (b) OER, and (c) bifunctional overpotentials Bayesian error estimation. The blue ellipsis and red lines show the covariance of error and standard deviation in each energy, respectively. The errors are calculated with respect to the flat surface in the CoCo model.

Results of nudged elastic band calculations Nudged elastic band (NEB) calculations were performed using ASE 3.22.1.[S2] The complete NEB results are presented in Figure S6.

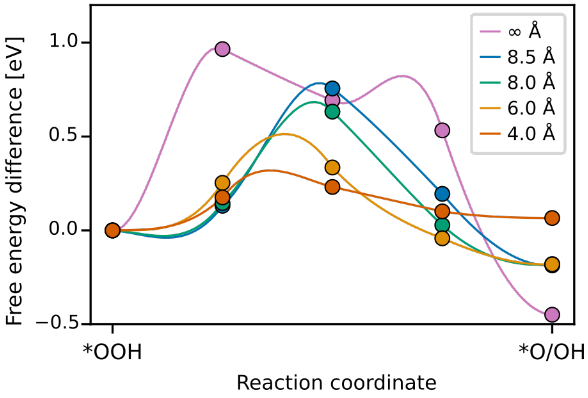

Figure S6: Results of nudged elastic band calculations for oxygen dissociation.

DFT calculated energies

Table S1: DFT calculated energies (in electronvolts) for adsorption intermediates.

Model	r	E_{surf}	E_{OOH}	$E_{\text{O/OH}}$	$E_{\text{OH/OH}}$	E_{OH}	ΔG_{OOH}	$\Delta G_{\text{O/OH}}$	$\Delta G_{\text{OH/OH}}$	ΔG_{OH}
CoCo	∞	−593.21	−607.63	−608.08	−613.21	−603.15	4.08	3.61	2.43	0.98
CoCo	8.5	−592.32	−606.72	−606.91	−612.17	−602.14	4.09	3.88	2.58	1.09
CoCo	8.0	−592.23	−606.62	−606.80	−612.07	−602.03	4.11	3.91	2.60	1.12
CoCo	6.0	−591.57	−605.84	−606.02	−611.33	−601.36	4.23	4.03	2.68	1.13
CoCo	4.0	−589.64	−603.69	−603.62	−608.68	−599.27	4.44	4.49	3.40	1.29
CoNi	∞	−592.17	−606.68	−606.41	–	−602.20	3.98	4.24	–	0.88
CoNi	8.0	−591.18	−605.63	−604.97	–	−601.08	4.05	4.68	–	1.03
CoNi	6.0	−590.67	−605.06	−604.38	–	−600.53	4.10	4.76	–	1.06
CoNi	4.0	−588.61	−602.75	−601.95	–	−598.36	4.35	5.14	–	1.16

References

(S1) Enkovaara, J.; Rostgaard, C.; Mortensen, J. J.; Chen, J.; Dułak, M.; Ferrighi, L.; Gavnholt, J.; Glinsvad, C.; Haikola, V.; Hansen, H. A.; Kristoffersen, H. H.; Kuisma, M.; Larsen, A. H.; Lehtovaara, L.; Ljungberg, M.; Lopez-Acevedo, O.; Moses, P. G.; Ojanen, J.; Olsen, T.; Petzold, V.; Romero, N. A.; Stausholm-Møller, J.; Strange, M.; Tritsaris, G. A.; Vanin, M.; Walter, M.; Hammer, B.; Häkkinen, H.; Madsen, G. K. H.; Nieminen, R. M.; Nørskov, J. K.; Puska, M.; Rantala, T. T.; Schiøtz, J.; Thygesen, K. S.; Jacobsen, K. W. Electronic Structure Calculations with GPAW: A Real-Space Implementation of the Projector Augmented-Wave Method. *J. Phys.: Condens. Matter* **2010**, *22*, 253202.

(S2) Larsen, A. H.; Mortensen, J. J.; Blomqvist, J.; Castelli, I. E.; Christensen, R.; Dulak, M.; Friis, J.; Groves, M. N.; Hammer, B.; Hargus, C.; Hermes, E. D.; Jennings, P. C.; Jensen, P. B.; Kermode, J.; Kitchin, J. R.; Kolsbjerg, E. L.; Kubal, J.; Kaasbjerg, K.; Lysgaard, S.; Maronsson, J. B.; Maxson, T.; Olsen, T.; Pastewka, L.; Peterson, A.; Rostgaard, C.; Schiøtz, J.; Schütt, O.; Strange, M.; Thygesen, K. S.; Vegge, T.; Vilhelmsen, L.; Walter, M.; Zeng, Z.; Jacobsen, K. W. The atomic simulation environment—a Python library for working with atoms. *J. Phys.: Condens. Matter* **2017**, *29*, 273002.

(S3) Hammer, B.; Hansen, L. B.; Nørskov, J. K. Improved Adsorption Energetics within Density-Functional Theory Using Revised Perdew-Burke-Ernzerhof Functionals. *Phys. Rev. B* **1999**, *59*, 7413–7421.

(S4) Wan, H.; Østergaard, T. M.; Arnarson, L.; Rossmeisl, J. Climbing the 3D Volcano for

the Oxygen Reduction Reaction Using Porphyrin Motifs. *ACS Sustain. Chem. Eng.* **2019**, *7*, 611–617.

(S5) Calle-Vallejo, F.; Martínez, J. I.; Rossmeisl, J. Density Functional Studies of Functionalized Graphitic Materials with Late Transition Metals for Oxygen Reduction Reactions. *Phys. Chem. Chem. Phys.* **2011**, *13*, 15639.

(S6) Caldeweyher, E.; Ehlert, S.; Hansen, A.; Neugebauer, H.; Spicher, S.; Bannwarth, C.; Grimme, S. A Generally Applicable Atomic-Charge Dependent London Dispersion Correction. *J. Chem. Phys.* **2019**, *150*, 154122.

(S7) Nørskov, J. K.; Rossmeisl, J.; Logadottir, A.; Lindqvist, L.; Kitchin, J. R.; Bligaard, T.; Jónsson, H. Origin of the Overpotential for Oxygen Reduction at a Fuel-Cell Cathode. *J. Phys. Chem. B* **2004**, *108*, 17886–17892.

(S8) Mortensen, J. J.; Kaasbjerg, K.; Frederiksen, S. L.; Nørskov, J. K.; Sethna, J. P.; Jacobsen, K. W. Bayesian Error Estimation in Density-Functional Theory. *Phys. Rev. Lett.* **2005**, *95*, 216401.

(S9) Bagger, A.; Ju, W.; Varela, A. S.; Strasser, P.; Rossmeisl, J. Single site porphyrine-like structures advantages over metals for selective electrochemical CO2 reduction. *Catal. Today* **2017**, *288*, 74–78.
